# Editorial: WT1 in Development and Disease

**DOI:** 10.3389/fcell.2022.969100

**Published:** 2022-07-08

**Authors:** You-Ying Chau, Ofelia M. Martínez-Estrada

**Affiliations:** ^1^ Centre for Cardiovascular Science, Queen’s Medical Research Institute, University of Edinburgh, Edinburgh, United Kingdom; ^2^ Celltec-UB, Department of Cell Biology, Physiology, and Immunology, Faculty of Biology, University of Barcelona, Barcelona, Spain; ^3^ Institute of Biomedicine (IBUB), University of Barcelona, Barcelona, Spain

**Keywords:** WT1, cardiac interstitial cells, choroid plexus, heart development, visceral adipose tissue, anemia, cancer

WT1 transcription factor (*WT1*) is a fascinating gene identified in 1990 as a gene involved in the development of a subset of Wilms’ tumors, a form of pediatric kidney cancer. Although it was first investigated in oncology research, the data generated in recent decades have revealed its natural role in the regulation of organ development (Hastie, 2017). Interestingly, recent studies have also highlighted its role in the maintenance and repair of several other tissues (Chau et al., 2011; Hastie, 2017).

In mammals, the *WT1* gene contains 10 exons, and multiple isoforms can be produced by combinations of alternative transcription start sites, translation start sites, alternative splicing, and RNA editing. WT1 has been demonstrated to function both as a transcriptional activator and as a repressor. Furthermore, new studies also support its post-transcriptional functions *via* RNA interactions (Bharathavikru et al., 2017; Hastie, 2017).

WT1 is abundantly expressed in progenitor cells during embryonic development. However, its expression is downregulated toward the end of gestation, and few differentiated cell types derived from these progenitors express high levels of WT1 in the adult (Hastie, 2017). Since the early characterization of the *Wt1KO* mouse model, it has been known that WT1 is required for the development of several tissues and organs (Kreidberg et al., 1993; Hastie, 2017). In fact, *Wt1KO* mice display agenesis of the kidney, gonad, and spleen, exhibit congenital diaphragmatic hernia (CDH), and lung hypoplasia (Hastie, 2017). The mid-gestation lethality observed in *Wt1KO* mice has been attributed to the cardiovascular defects observed in this mouse model (Moore et al., 1999; Hastie, 2017).

Over the last decade, the generation of conditional *Wt1KO* mouse models has made it possible to bypass the early lethality of conventional *Wt1KO* mice and selectively delete *Wt1* in a cell-specific and time-dependent manner (Gao et al., 2006; Martinez-Estrada et al., 2010; Berry et al., 2015). In combination with new reporter mouse models, these new tools have had a profound impact on WT1 biology, the phenotypic anomalies linked to *Wt1* deletion have been expanded, and new tissues and phenotypes have been identified (Hosen et al., 2007; Zhou et al., 2008; Wessels et al., 2012; Hastie, 2017).

The current Research Topic for Frontiers in Cell and Developmental Biology comprises five contributions, including three original research articles and two mini-reviews that highlight relevant new aspects of WT1 biology.

In the first article published on this Research Topic, “*Deletion of the Wilms’ tumor suppressor gene in the cardiac troponin-T lineage reveals novel functions of WT1 in heart development*,” Díaz del Moral et al. report the transient expression of WT1 in a population of embryonic cardiomyocytes. Interestingly, despite this transient expression, its conditional ablation using a Tnnt*2* ^*Cre*^ driver caused abnormal heart development. The authors report a series of heart defects including abnormal sinus venosus and atrium development, lack of pectinate muscles, and thin ventricular myocardium. After analyzing the phenotype of the mutant mice, they conducted a transcriptomic analysis which demonstrated that *Wt1* deletion using Tnnt*2* ^*Cre*^ driver modified the transcriptomic signature of the embryonic heart. A Gene Ontology (GO) functional enrichment analysis suggested that both calcium ion regulation and modulation of potassium channels were significantly altered in the mutant hearts. Use of the conditional *Wt1KO* mouse model has enabled Díaz del Mora et al. to demonstrate that WT1 in Tnnt2^Cre^ lineage cardiomyocytes is required for normal cardiac development, further expanding the role of WT1 in the developing heart.

The heart is also the subject of another research article included in this Research Topic. Cardiac interstitial cells (CICs) form a dynamic and heterogeneous population of cells. In the article entitled “*Dynamic epicardial contribution to cardiac interstitial c-Kit and Sca1 cellular fractions*,” Pogontke et al. report using *Wt1* reporter mouse models to characterize cardiac interstitial populations of cells in the developing heart. Using a constitutively active Wt1Cre mouse model (Wt1^Cre^), the authors show that around 50% of cardiac c-Kit^POS^ cells are derived from the *Wt1* lineage at E15.5 and that the number of this subpopulation decreases during embryonic development. They also found that the percentage of Sca1^POS^ cells within the *Wt1* lineage increases postnatally, and demonstrated that the majority of cardiac *Wt1*-lineage derived endothelial cells and fibroblasts express Sca1 in the adult heart. Furthermore, they used an inducible *Wt1*-lineage tracing model (Wt1Cre^ERT2^) to rule out the novo postnatal appearance of cardiac Sca1^Pos^ *Wt1*-lineage derived cells.

In addition to mouse as a model organism, zebrafish has also been very valuable in deciphering new functions of Wt1 in development and repair (Hastie, 2017). Another original research article included in this Research Topic is the article entitled “*The Wilms Tumor Gene wt1a Contributes to Blood-Cerebrospinal Fluid Barrier Function in Zebrafish*” by Hopfenmüller et al., who have discovered new expression domains for Wt1a in the dorsal hindbrain, the caudal medulla, and the spinal cord of zebrafish. Marker analysis identified wt1a -expressing cells from the dorsal hindbrain as ependymal cells of the choroid plexus in the myelencephalic ventricle. The authors analyzed wt1a mutant larvae and demonstrate that *wt1a* is required for proper choroid plexus formation and function. Thus, the use of zebrafish as a model has enabled Hopfenmüller et al. to determine that Wt1a contributes to the barrier properties of the choroid plexus, revealing an unexpected role for Wt1 in the zebrafish brain.

In addition to these three research articles, our Research Topic also includes two mini-reviews of the literature on WT1 in adipose tissue and the link between WT1, anemia, and cancer in chronic kidney disease. In the article entitled “*WT1 in Adipose Tissue: From Development to Adult Physiology*,” Kirschner and Scholz et al. review the role of WT1 in the development of visceral white adipose tissue (WAT) and its function as a regulator of visceral white adipose identity, while in the article entitled “*WT1: The Hinge Between Anemia Correction and Cancer Development in Chronic Kidney Disease*,” Lee et al. discuss the link between HIF, WT1, anemia correction, and cancer.

With this Research Topic, we have expanded our understanding of WT1 functions, especially in heart and brain development. We acknowledge all authors who contributed to this Research Topic, and we hope that this collection will inspire the community to conduct further research into this complex but fascinating gene.
